# Will your child take care of you in your old age? Unequal caregiving received by older parents from adult children in Sweden

**DOI:** 10.1007/s10433-023-00755-0

**Published:** 2023-04-03

**Authors:** Isabelle von Saenger, Lena Dahlberg, Erika Augustsson, Johan Fritzell, Carin Lennartsson

**Affiliations:** 1 Aging Research Center, Karolinska Institutet and Stockholm University, Stockholm, Sweden; 2 School of Health and Welfare, Dalarna University, Falun, Sweden; 3 Swedish Institute for Social Research, Stockholm University, Stockholm, Sweden

**Keywords:** Intergenerational caregiving, Gender, Social class, Socioeconomic inequalities, Informal caregiving, Sweden

## Abstract

Intergenerational family care provided to older parents by adult children is growing and differs based on gender and socioeconomic status. Few studies consider these elements in relation to both the parent and their adult child, and little is known about the number of care tasks received even though those providing intensive levels of care are at risk of experiencing adverse consequences in their lives. This study uses data from the nationally representative 2011 Swedish Panel Study of Living Conditions of the Oldest Old (SWEOLD) and includes child-specific information from parents aged 76 years and above. Analyses used ordinal logistic regression and are presented as average marginal effects and predictive margins. Results show that parents in need of care report that one-third of all adult children in the sample provide care to three out of five of them. The care is most often non-intensive, yet nearly one in ten of all children provide more intensive care of two or more tasks. When adjusting for dyad characteristics as well as geographic proximity, results show adult–child gender differences where parents receive more care from manual-working-class daughters than manual-working-class sons. Overall, manual-working-class daughters are most commonly reported as carers among adult children, and they are particularly overrepresented in providing intensive care. We conclude that gender and socioeconomic inequalities exist among care receivers’ adult children, even in a strong welfare state such as Sweden. Knowledge about levels and patterns of intergenerational care have important implications for how to reduce unequal caregiving.

## Introduction

Family care remains the most common source of care for older adults and exceeds formal care provisions from the welfare state. In Sweden, where formal care is comprehensive, family care is estimated to be three times as common (Wimo et al. 2020), with adult children providing around half of the family care for older adults (National Board of Health and Welfare 2012). Generally, family care has increased in recent decades (Wolff et al. 2017). This increase has been more evident in Sweden compared to other Nordic countries (Szebehely and Meagher 2018). In caregiving studies, it is important to separate spouses from adult children, as they reflect fundamental differences in both the experiences and consequences of caregiving (Qualls 2021). Caregiving can be a positive practice; however, children providing intensive care more often tend to have poorer health, lower labour market participation and fewer financial resources (Bastawrous et al. 2015; Szebehely et al. 2014).

Parental caregiving has been shown to differ by gender and socioeconomic position of both parents and their adult children (Wong et al. 2020). However, in Sweden, studies on socioeconomic differences in caregiving have mostly focused on parental social position and not that of the adult child. In addition, to our knowledge, few studies internationally and in Sweden consider both the gender and socioeconomic position of the adult child when assessing subgroup differences in parent–child caregiving intensity. Even less is known about these care patterns when controlling for parental characteristics and geographic proximity. Caregiving inequalities are particularly important, as informal caregiving may enhance health and financial inequalities. This study examines the distribution and intensity of caregiving received by older parents from each individual adult child, with a specific focus on the gender and social class differences of the children.

### Intergenerational informal care

*Informal care* refers to unpaid care provided by family members, neighbours or friends of a person needing support due to disability, long-term illness, or old age. It can range from personal care, such as personal hygiene or showering, often considered intensive and demanding for the provider, to lower-intensity practical care such as support with household chores, shopping, or transport (Szydlik 2016). The range of care types received is important for the health and well-being of the recipient (Li and Song 2019).

It can be said that Sweden and the neighbouring Nordic countries relieve families of care obligations through relatively extensive public care services. Swedish law states that care provided by family members should be voluntary and that the state is to be the primary source of care when needed (Prop.^2008^/09:82 2008). This prioritisation is supported by both older adults and their family members (Szebehely and Trydegård 2007) as well as the Swedish population (Svallfors 2011). Nevertheless, studies show that adult children more often provide care, however, less intensive, in generous welfare state countries compared to countries where family obligations are greater and there is less public spending on care for older adults (Deindl and Brandt 2011; Verbakel 2018). When adult children are relieved from intensive care tasks, more children seem to provide less intensive care to their parents (Saraceno 2010). Despite the significance of the parent–child care relationship, it has received less attention in the caregiving literature in Sweden, than for example spousal care (National Board of Health and Welfare 2020; Ulmanen 2015).

### Gender and socioeconomic inequalities

International findings consistently show that daughters are more likely to care for an older parent compared to sons (Wong et al. 2020), especially if the parent is a mother (Grigoryeva 2017; Silverstein et al. 2006; Szydlik 2016). Findings in Sweden are less clear and vary depending on the assessment of caregiving. Gender differences tend to be insignificant when considering whether care has been provided at all, or at least monthly (Szebehely et al. 2014; von Essen and Svedberg 2020). However, there are gender differences regarding the type of care and care intensity; daughters are more likely to provide intensive care, for example, personal care (Kridahl and Duvander 2021) together with other care tasks. Sons, on the other hand, tend to provide practical care (Jegermalm 2006). To accurately capture gender differences in caregiving, it is important to incorporate as wide a range of care tasks as possible, including those where sons are the most prevalent caregivers (Ulmanen 2016).

Universalism is a cornerstone in the Swedish welfare system, e.g. publicly funded care for older adults should be available to all as needed, regardless of an individual’s financial or family resources (Sipilä 2019). However, care provided by the state has undergone a process of deinstitutionalisation and marketisation, having various implications for the lives of older adults and their families (Rostgaard et al. 2022). Older adults needing care appear to arrange their care provision depending on their socioeconomic status, especially if they do not qualify for formal services. Previous research in both Europe and Sweden has shown that those with lower socioeconomic resources receive more care from family members, than those with higher socioeconomic resources, who are more likely to enlist private-sector providers (van Groenou et al. 2006; Sarasa Urdiola and Billingsley 2008; Ulmanen 2015). In Sweden, such market solutions have also been fostered by the introduction of tax relief for many domestic services.

Caregiving to any family member seems to be more common when caregivers are from low socioeconomic groups, especially when the care is more intensive (Carmichael et al. 2010; Tough et al. 2019) and provided by a spouse (Glaser and Grundy 2002). Socioeconomic differences in parental caregiving show mixed results. In a comparative European study (Brandt 2011) all forms of caregiving were more common among adult children with higher education than lower, while the opposite was found in another comparative European study (Sarasa Urdiola and Billingsley 2008). However, the latter did not find any such differences among the Nordic countries. One explanation for the likelihood of higher educated children providing more care could be related to the association between education and health (Zimmerman and Woolf 2014). Good health is a prerequisite for providing intensive care, and higher education might correlate more in countries with weaker welfare states and educational systems. The non-differential socioeconomic patterns of the Nordic countries have been confirmed in both Norway (Gautun and Hagen 2010) and Sweden (Jegermalm and Grassman 2012; von Essen and Svedberg 2020). Even if this socioeconomic caregiving perspective has been less researched in the Nordic countries (Ulmanen 2022), there are indications of care intensity being an important factor for distinguishing socioeconomic differences among caregiving adult children.

Research addressing both gender and socioeconomic differences in family caregiving indicates that gender differences can be modified by education. In the UK, women with lower education were most likely to provide intensive care to a parent (Henz 2021) and in Japan, they were most likely to be the primary caregiver (Tokunaga and Hashimoto 2017). In Sweden, parents with a lower socioeconomic status were more likely to receive care from a daughter than a son (Ulmanen and Szebehely 2015). However, the associations in Sweden seem to vary across specified care relationships. For example, one study found that higher-educated women were slightly more likely to be caregivers to any family member than women with lower education (Ulmanen 2022), while the opposite was found when focusing on adult child characteristics in parental caregiving during the 1990s (Winqvist 1999). Daughters with lower educational levels were more likely to provide intensive care such as household-related tasks and personal care to an older parent, while sons with lower educational levels would provide less intensive care such as repairs and gardening. No gender differences were found among highly educated children, which was explained by a more equal distribution of care tasks provided. To our knowledge, this gender and socioeconomic interaction of adult children have not been investigated further among care-receiving older parents in Sweden and even less is known when taking care intensity, parental characteristics and geographic proximity into account.

### Geographic proximity and other potential covariates

Living close to an adult child facilitates care receipt through more efficient use of time and reduced travel costs. This is particularly significant for regular and demanding care (Pillemer and Suitor 2013; Wong et al. 2020). Decisions to move closer to a child or a parent can be determined by a prospective need to provide care for both older and younger generations (Pettersson and Malmberg 2009). Mothers are more likely to live closer to an adult child than fathers, as are parents with lower levels of education (Choi et al. 2015; Lennartsson 2001). Chan and Ermisch’s (2015) study of the UK found no gender difference between children in geographic proximity to parents, although more educated children tended to live further away.

Parental age is also an important factor in care needs. Functional limitations and health problems become more frequent and permanent with age, especially beyond the age of 80 (Fors et al. 2022; Nilsen et al. 2019). Time availability from the caregivers’ perspective is also important, as adult children at work provide less care (Wong et al. 2020).

This study adds to previous research by assessing the prevalence, intensity and distribution of informal caregiving received by older parents from individual adult children, while taking parental and child characteristics as well as geographic proximity into consideration. The focus is on the gender and socioeconomic differences of adult children providing care to older parents in need. Inequalities in formal and informal care reception among older adults are well-established. However, it is also crucial to understand the overall distribution and possible subgroup differences in the caregiving of older parents by adult children (Qualls 2021; Tokunaga and Hashimoto 2017; Ulmanen and Szebehely 2015). Only then can we identify potentially vulnerable groups of adult children providing care and risking adverse health and financial outcomes and provide social policy measures against increasing inequalities in family caregiving.

## Aim

This study aims to describe the distribution of informal care received by older parents from adult children with a specific focus on gender and social class differences among children. The study asks:

1. What portion of adult children are parents reporting as providing care to them and how are care tasks distributed among the children?
2. Does the distribution of care tasks differ by adult children’s gender and social class as reported by older parents?
3. To what extent can these differences be explained by parental characteristics and geographic proximity?

## Data and methods

### Design and participants

**Fig. 1:**
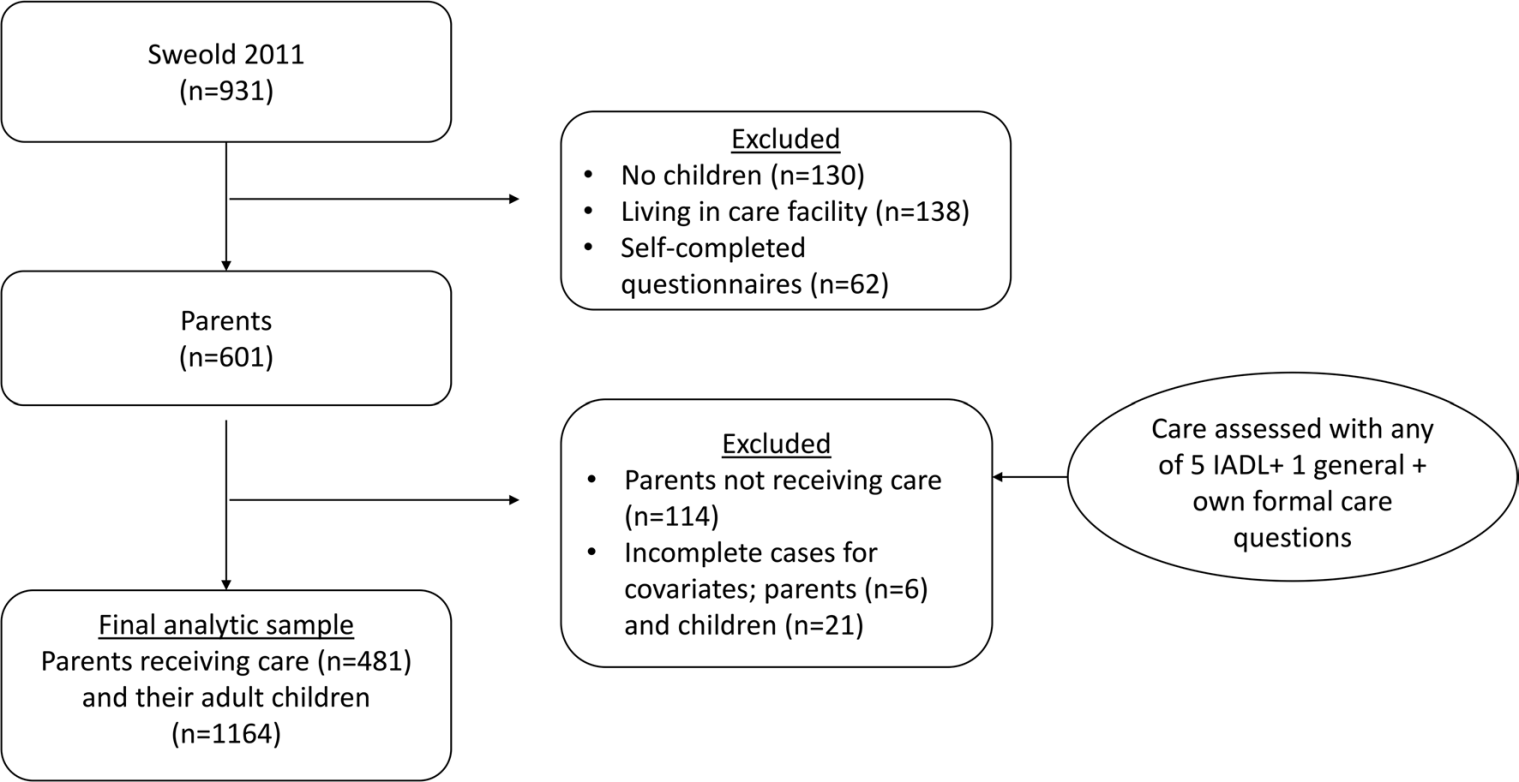
Flow chart of the final analytic sample with parents (*n* = 481) and their adult children (*n* = 1164)

Data were taken from the 2011 *Swedish Panel Study of Living Conditions of the Oldest Old* (SWEOLD), a randomly sampled national survey conducted continually since 1992 comprising 931 people in 2011. It is a nationally representative sample of people born between 1909 and 1934 aged 76 and above. The response rate was 86.2% and interviews were conducted face to face, with the option of taking place over the telephone. If a person was unable to participate due to issues such as dementia or frailty, an indirect interview was performed with a close relative or healthcare worker (Lennartsson et al. 2014). The sample included only those with adult children who needed care, indicated by being a care recipient. The following types of care being received formed the eligibility inclusion: help buying and/or preparing food, cleaning, help with personal hygiene, providing transport, etc. (see dependent variable), general household or personal care, and formal care. People living in care facilities and responding via self-completion questionnaires (as they did not receive any questions about who helped them) were excluded. After omitting incomplete cases for covariates, the final analytical sample consisted of 481 older parents with child-specific data on 1164 adult children (see Fig. 1). Informed verbal consent was obtained prior to each interview. Ethical approval was provided by the Regional Ethical Review Board in Stockholm (2010/403–31/4) and Ethical Review Agency (2019-06324).

### Dependent variable

The dependent variable concerns the number of care tasks received by parents from each adult child. Five survey questions were used and incorporated a range of care tasks, all defined as practical care, including the more intensive personal care task of bathing and/or showering.

The first four questions were: Do you usually buy food by yourself? Do you usually prepare food by yourself? Do you usually clean the house by yourself? and Do you bath/shower yourself? The response alternatives were: ‘Yes, unaided’, ‘Yes, with help’ and ‘No, not at all’. The latter two responses were followed with the question: Who usually helps you with…?. Multiple answers were possible, such as cohabitant, daughter, son, and formal care services, and when the answer was a child, each specific child was linked by their name to create a child–parent dyad.

The last question, here referred to as ‘transportation, etc.’, was: Over the last 12 months, have you received any help from relatives or friends (not living with you) with any of the following: repairs or maintenance; gardening, personal finance, buying clothes or other items, being driven somewhere (response options: Yes, No). ‘Yes’ answers were followed with the question: Who usually helps you? Multiple answers were possible such as daughter, son, female/male relative, or another person. If the answer was a child, that specific child was linked by their name. Receiving help with transportation was the most common response and a requirement for inclusion in this care category. Other tasks within the category ranged from 34% (repairs and maintenance) to 50% (buying clothes or other items), with a mean of 2.76 tasks mentioned, and a standard deviation of 1.21. The tasks included in the transportation, etc., group are generally of lower intensity (Ulmanen 2016).

The final dependent variable was represented by four groups: no care received; one care task; two care tasks; three or more care tasks received from the adult child. Relatively few children were reported to provide more than three care tasks, so the last category represents three to five care tasks (see Table 2).

### Independent variables

Gender and social class of the adult children were the main independent variables of interest. Social class was assessed using the official Swedish socioeconomic classification (SEI), which is based on and similar to several dimensions of the internationally established Erikson–Goldthorpe–Portocarero (EGP) classification (Erikson and Goldthorpe 1992). The occupation and position in the labour market of the respondent are the foundation for SEI (Andersson et al. 1981).

Given that adult child occupation information was provided by parents, the possibility to distinguish between a wider range of occupations was more restricted compared to the parent. Adult child social class was represented by four groups: manual workers; non-manual workers, self-employed and farmers, and unclassified.

Parental gender, social class, and geographic proximity formed other independent variables. Social class was measured using the same classification as for children. Since the occupation of both the respondent and the living or deceased spouse was known, a household class position was assigned to the parent, assuming that some positions dominate over others in terms of values, attitudes, and behavioural patterns (Erikson 1984). Parental household class was represented by four groups: manual workers, lower non-manual workers, intermediate and higher non-manual workers, and self-employed and farmers. Geographic proximity was represented by three categories: less than 20 km; 21 to 100 km; and over 100 km.

Control variables included the adult child’s labour market activity represented by working; retired; other (where ‘other’ includes, e.g. the unemployed persons and students), parental age and if the parent was living alone.

## Analytic approach

Descriptive statistics were presented for all study variables. Bivariate analyses were performed to determine differences between independent variables and care intensity using Chi^2^ tests. Ordinal logistic regression models were used to study the association between gender and social class of adult children and receiving increasing numbers of care tasks. Estimates were presented as average marginal effects (AME) and 95% confidence intervals to enable comparisons across models (Mood 2010; Williams 2020). AME can be interpreted as the average difference in probability (0–1) of the outcome depending on the value of the independent variable. Analyses were stratified by social class of children, as social class may modify gender differences in caregiving. Finally, we use predictive margins (PM) to present the probability of receiving informal care across the different caregiving groups by gender and social class of children while holding all other variables constant, including an interaction term of gender and social class of children. PM facilitates the interpretations of the results compared to regression coefficients, especially when presenting group differences in the presence of interaction terms (Graubard and Korn 1999). Significant levels were set at *p* < 0.05. Since the regressions are based on our constructed adult child population, the unit of analysis is not independent (several children can share the same parent). Therefore, we perform our statistical tests with robust standard errors adjusted for clustering. Analysis was performed using weights to compensate for unequal probability to be included in the sample depending on gender and age (85+) in the parental sample. Data were analysed using Stata 17.0 for Windows.

## Results

Table 1 presents the baseline characteristics of the parents and their adult children. Most parents received a combination of care from children and others, with ‘others’ including partners, relatives, friends, formal, or private care. A minority of parents, 15%, only received care from their children. Around half of the parents were 85 years or older and more than half lived alone. Eight out of ten children were employed, and their mean age was 54 years. More than half of the children lived less than 20 km from their parent.

**Table 1:** Baseline characteristics of the study sample

Parents	*n*	%
Care receipt		
From adult children only	74	15.38
From adult children and others	214	44.49
From others only	193	40.12
Gender		
Mothers	243	50.52
Fathers	238	49.48
Household class		
Manual workers	116	24.12
Lower non-manual workers	73	15.18
Intermediate/higher non-manual workers	147	30.56
Self-employed/farmers	145	30.15
Age groups		
76–79	98	20.37
80–84	133	27.65
85+	250	51.98
Living alone	260	54.05
	Mean (SD)	Range
Number of adult children	3.1 (1.27)	1–10

Adult children		
Gender		
Daughters	588	50.49
Sons	576	49.51
Own class		
Manual workers	309	26.57
Non-manual workers	528	45.33
Self-employed/farmers	184	15.81
Unclassified	143	12.28
Labour market activity		
Working	968	83.18
Retired	106	9.12
Other	90	7.70
Distance between adult child and parent		
Less than 20 km	607	52.14
21–100 km	221	18.97
More than 100 km	336	28.89
	Mean (SD)	Range
Adult child age in years	53.96 (7.54)	18–78

Parents in need of care (*n* = 481) and their adult children (*n* = 1164)

^a^Parental characteristics not weighted

**Fig. 2:**
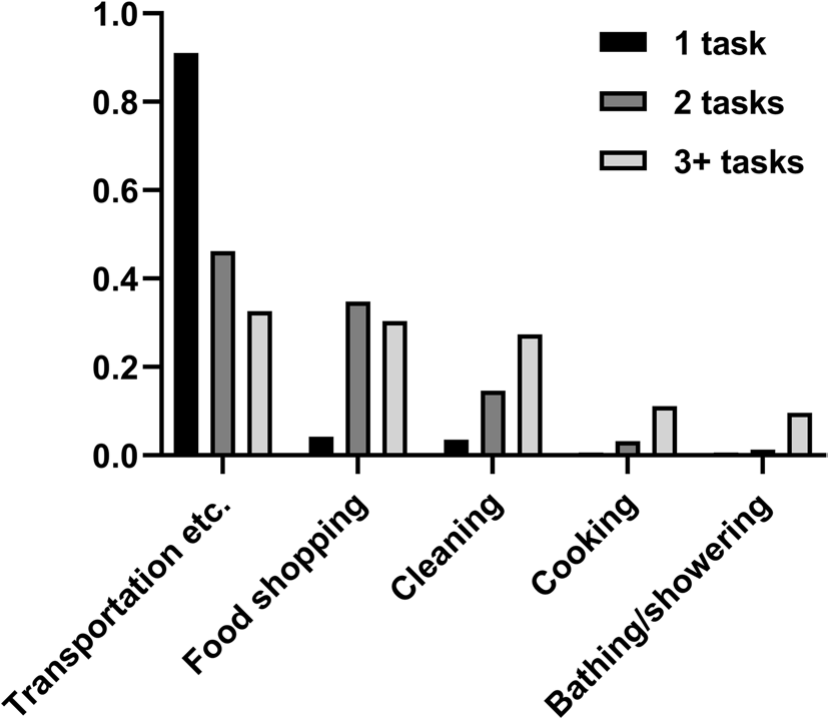
Average distribution of care types such as transportation, etc., food shopping, and cleaning, received by older parents by three caregiving groups, defined as the number of tasks provided by adult children. Per cent. (*n* = 436)

Figure 2 shows how care types were distributed within three caregiving groups. Parents reported that nine out of ten children who provided a single care task were helping them with transportation, etc. For the two care task group, the distribution became more even; transportation, etc., still dominated (47%) followed by food shopping (34%) and cleaning (15%). Finally, in the three or more care task group, differences between transportation, etc. (33%), food shopping (30%), and cleaning (27%) diminished, whereas cooking (11%) and bathing/showering (10%) became more prominent. Hence, providing more care tasks means providing a more diverse and intensive type of caregiving compared to those providing fewer care tasks. The differences across the groups can therefore be interpreted as differences in care intensity.

## Bivariate analyses

The first column in Table 2 shows that parents in need reported that around one-third of all adult children in the sample provided some kind of care to them. Thus, parents in need of care do not receive care from two-thirds of the children in the sample, but from other sources. Care was slightly more commonly received from daughters than sons, as was care received from manual workers compared to other social classes. The first row in Table 2 shows that one in four children was reported to provide one care task, whereas two (5%) and three or more tasks (3%) were less common. Sons were more often reported to provide one task, while daughters were almost twice as prevalent among two-task providers and five times as prevalent among three-or-more-task providers. Patterns of care distribution also varied across social classes. Manual workers were more often reported to provide one (29% vs. 23%) and two tasks (9% vs. 4%) than non-manual workers, while no difference was found in the most care-intensive group. Self-employed and farmers followed the general pattern of non-manual workers in terms of being less represented in all caregiving groups compared to manual workers. However, non-manual workers were least commonly reported as performing one task than all other social classes.

**Table 2:** Baseline characteristics of the adult child study sample by informal caregiving group as reported by older parents (*n* = 1164)

Caregiving group	No care *n* = 763, (65.53%)	1 care task *n* = 300, (25.76%)	2 care tasks *n* = 63, (5.44%)	3+ care tasks *n* = 38, (3.27%)	*p* value
*Adult children*					
Gender					< 0.001
Sons	384 (66.65)	163 (28.30)	22 (3.78)	7 (1.27)	
Daughters	379 (64.42)	137 (23.28)	42 (7.07)	31 (5.23)	
Own class					0.034
Manual workers	173 (58.08)	86 (28.87)	27 (9.12)	12 (3.93)	
Non-manual workers	371 (67.91)	127 (23.27)	24 (4.35)	24 (4.48)	
Self-employed/farmer	127 (69.75)	48 (26.14)	7 (3.96)	0 (0.15)	
Unclassified	93 (67.41)	38 (27.76)	5 (3.42)	2 (1.41)	
Labour market activity					< 0.001
Working	603 (66.22)	242 (26.56)	42 (4.59)	24 (2.63)	
Retired	92 (53.73)	40 (23.37)	21 (12.23)	18 (10.68)	
Other	59 (71.98)	16 (20.01)	5 (6.54)	1 (1.47)	
Distance to parent					< 0.001
Less than 20 km	308 (51.20)	214 (35.49)	50 (8.26)	30 (5.05)	
21–100 km	164 (72.17)	51 (22.50)	10 (4.37)	2 (0.96)	
More than 100 km	292 (87.02)	35 (10.35)	4 (1.05)	5 (1.59)	
*Parents*					
Gender					< 0.001
Fathers	415 (72.56)	135 (23.59)	18 (3.10)	4 (0.75)	
Mothers	356 (60.18)	162 (27.41)	43 (7.22)	31 (5.19)	
Household class					< 0.001
Manual workers	174 (63.16)	80 (28.68)	14 (5.24)	8 (2.73)	
Lower non-manual workers	88 (57.30)	53 (34.28)	9 (5.62)	4 (2.80)	
Intermediate/higher non-manual workers	252 (75.83)	69 (20.86)	7 (2.21)	4 (1.10)	
Self-employed/farmers	246 (61.10)	99 (24.59)	34 (8.47)	24 (5.85)	
Age groups					< 0.001
76–79	182 (73.98)	53 (21.54)	6 (2.44)	5 (2.03)	
80–84	214 (64.65)	95 (28.70)	14 (4.23)	8 (2.42)	
85+	350 (59.63)	152 (25.98)	54 (9.19)	31 (5.21)	
Living alone					< 0.001
Yes	358 (56.68)	188 (29.78)	56 (8.91)	29 (4.63)	
No	399 (75.07)	114 (21.44)	9 (1.69)	10 (1.80)	

Row percentage. Statistical significance between groups (Chi^2^): *p < 0.05

Older adults more commonly received intensive caregiving from children who had retired. Nevertheless, seven per cent of all reported children (4.6% + 2.6%), provided two or more care tasks on a regular basis while still working. Children living close to a parent were also reported to provide a higher number of care tasks; however, more than one in ten of those living the furthest away still provided one or more care tasks.

In terms of the parental characteristics across caregiving groups, our data show that mothers reported more care tasks received by their adult children than fathers. Parents in the intermediate or higher non-manual household class reported that their adult children provided the least care compared to all other social classes. Parents who had been self-employed or farmers reported receiving the most intensive care from their adult children. Intensive caregiving was most common for parents aged 85 and older. Parents who lived alone reported a higher share of caregiving by children in all caregiving groups.

## Multivariate analyses

Table 3 presents the average difference in the probability of informal caregiving across each caregiving group regarding adult children’s gender and social class. When controlling for children’s labour market activity, Model 1 shows no gender differences. Non-manual workers and self-employed and farmers were statistically significantly less likely to be reported as providing increasing care tasks than manual workers. The self-employed and farmers were 12.1 percentage points less likely than manual workers, while the equivalent for non-manual workers was 9.9 percentage points. Excluding child labour market activity made no important changes to these estimates (not shown). Adjusting for both child and parental characteristics and geographic proximity (model 2), the significant social class patterns weakened. Instead, gender differences strengthened, where daughters were 5.7 percentage points more likely than sons to be reported as providing any kind of care. Sensitivity analysis (not shown) revealed gender differences when including geographic proximity as a final covariate to the model even though parents tended to live further away from their daughters than sons. The social class differences in the first model were largely explained by geographic proximity since parents were living closer to children with manual occupations.

**Table 3:** Average marginal effects (AME) and 95% confidence intervals of the number of care tasks by adult children’s gender and social class as reported by older parents (*n* = 1164)

No. of care tasks	Model 1				Model 2			
	0	1	2	3+	0	1	2	3+
Gender								
Sons	Reference category				Reference category			
Daughters	− 0.044	0.028	0.010	0.006	− **0.057**	**0.034**	0.014	0.009
	(− 0.103, 0.014)	(− 0.009, 0.065)	(− 0.004, 0.023)	(− 0.003, 0.015)	**(**− **0.111, **− **0.003)**	**(0.003, 0.065)**	(− 0.000, 0.028)	(− 0.000, 0.019)
Social class								
Manual workers	Reference category				Reference category			
Non-manual workers	**0.099** **(0.022, 0.176)**	− **0.062** **(**− **0.110, **− **0.014)**	− **0.022** **(**− **0.042, **− **0.003)**	− **0.015** **(**− **0.028, **− **0.003)**	0.018 (− 0.049, 0.085)	− 0.010 (− 0.049, 0.029)	− 0.004 (− 0.021, 0.012)	− 0.003 (− 0.015, 0.008)
Self-employed/farmers	**0.121** **(0.029, 0.214)**	− **0.077** **(**− **0.136, **− **0.017)**	− **0.027** **(**− **0.049, **− **0.003)**	− **0.018** **(**− **0.033, **− **0.003)**	0.077 (− 0.001, 0.155)	− 0.047 (− 0.096, 0.001)	− 0.018 (− 0.036, 0.001)	− 0.012 (− 0.025, 0.001)

Model 1 adjusted for child labour market activity. Model 2 adjusted for child labour market activity and parental characteristics: gender, household social class, age, living alone as well as geographic proximity between parent and child. Statistically significant values in bold (*p* < 0.05). Unclassified social class not shown

We also analysed whether the association between adult children’s gender and care intensity was modified by their social class. Table 4 shows the full model (Table 3, Model 2) stratified by child social class. The results show that daughters in manual occupations were reported to perform significantly more care tasks than sons. There was a 16.2 percentage point lower probability that a daughter in the manual social class group would provide no care, and 3.1 percentage point higher probability of performing three or more tasks than sons. There were no significant gender differences among other social classes.

**Table 4:** Average marginal effects (AME) and 95% confidence intervals of the number of care tasks by adult children’s gender and stratified by their social class, as reported by older parents (*n* = 1164)

	0	1	2	3+
*Manual workers*				
Sons	Reference category			
Daughters	− **0.162** **(**− **0.262, **− **0.062)**	**0.071** **(0.031, 0.112)**	**0.059** **(0.014, 0.105)**	**0.031** **(0.002, 0.061)**
*Non-manual workers*				
Sons	Reference category			
Daughters	0.023 (− 0.053, 0.098)	− 0.013 (− 0.057, 0.031)	− 0.004 (− 0.018, 0.010)	− 0.005 (− 0.023, 0.012)
*Self-employed/farmers*				
Sons	Reference category			
Daughters	0.034 (− 0.094, 0.162)	− 0.026 (− 0.124, 0.072)	− 0.007 (− 0.037, 0.022)	− 0.000 (− 0.002, 0.001)

Adjusted for child labour market activity and parental characteristics, such as gender, household social class, age, living alone as well as geographic proximity between parent and child. Statistically significant values in bold (*p* < 0.05). Unclassified social class not shown

**Fig. 3:**
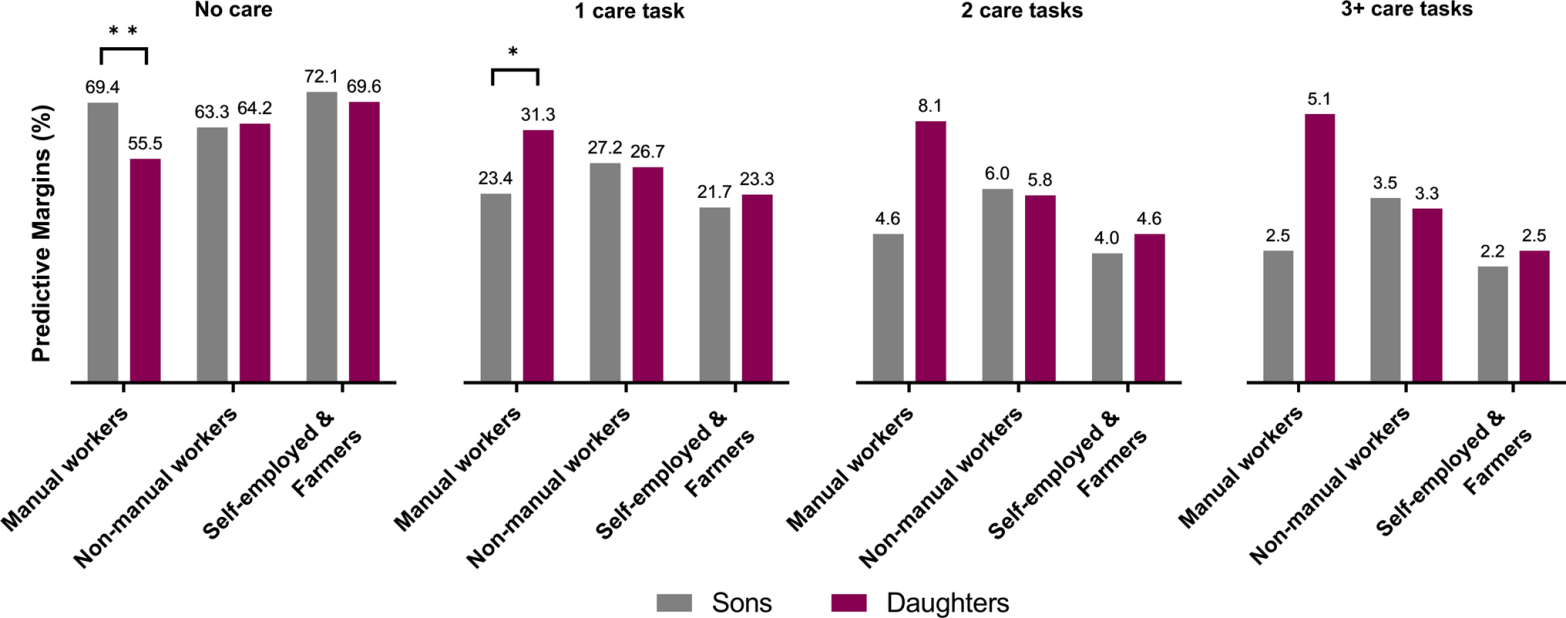
Predictive margins (PM) of belonging to different caregiving groups, as reported by older parents, by adult child gender and social class. The results have been adjusted for adult child and parental characteristics, geographic proximity including an interaction term of child gender and social class (*n* = 1164). Statistical significance between groups: *p < 0.05; **p < 0.01

Figure 3 shows the predictive margins (PM) from the ordinal logistic regression presented in Table 3 (Model 2), including a two-way interaction term between adult children’s gender and social class. The PM can be interpreted as the reported probability of belonging to each caregiving group for daughters and sons, respectively, depending on their social class, while holding other variables in the model constant. Results show that manual-working daughters were 44.5% likely to provide care, whereas manual-working sons were 30.6% likely. The relative difference of 8 percentage points between manual-working daughters and sons remained significant among those reported to perform one care task. Patterns across the more intensive care groups suggest an increase in this relative difference. Results showed no gender differences among the other social classes in any of the caregiving groups. Further analysis showed that adding formal care receipt of the parent as a covariate in this final model did not significantly change the estimates (not shown), which is a finding in itself. Overall, regardless of care intensity, daughters of manual occupations were more often reported to be caregivers than other adult children.

## Discussion

This study aims to describe the distribution of informal care received by older parents from adult children with a specific focus on these children’s gender and social class differences. Our study shows that 15% of parents receiving care had this provided only by their child. We also found that parents reported that 34% of the children in our sample provided care on a regular basis and that the care was generally of a non-intensive practical nature. Five per cent of children were reported as performing two tasks, usually a combination of transportation, etc., and one other practical care task, most commonly food shopping, or cleaning. Three per cent of all children were reported to provide three or more care tasks, thus being the most care-intensive group.

This confirms findings that indicate how it is the type of care received by older adults that reflects the relationship between welfare state design and caregiving patterns in a country—not only the proportion of adult children providing care (Saraceno 2010). Our study shows that the most common kind of care received by older parents from children was non-intensive, mirroring care needs that are seldom covered by formal care services, thus falling naturally on close family members such as adult children. The findings are therefore in line with the complementarity theory discussed by Litwak (1985), which claims that informal care complements formal care. The findings also agree with the notion of ‘crowding in and out’ (Szydlik 2016), where an extensive welfare state relieves (crowds out) children from intensive caregiving while increasing (crowds in) the need for non-intensive caregiving. However, this concept does not seem to fit all adult children. Ten per cent were reported to provide two or more care tasks, thereby performing a larger variety of task such as domestic and personal care tasks, which are also offered by formal care services.

This study shows that the receipt of caregiving and its intensity differ by adult children’s gender and socioeconomic status. There were also differences linked to parental characteristics and geographic proximity between children and parents. Compared to sons, daughters were reported to provide more intensive care by combining practical and sometime personal hygiene tasks with transportation, etc. Daughters were also reported to provide most care even when they lived further away than sons. That differences in social class weakened in the full model is partially explained by the diversity in distance between parents and children depending on social class belonging. While this study considers several aspects lacking in previous research, the findings about gender differences are in line with international research (Wong et al. 2020). In the Swedish context, results provide a more nuanced picture of gender differences in intergenerational old age care. This study highlights the importance of assessing care intensity and type of care provided (Szebehely et al. 2014; von Essen and Svedberg 2020) in combination with accounting for the dyad characteristics, specifically geographic proximity (Jegermalm 2006; Kridahl and Duvander 2021) for better understanding potential gender differences. The non-differential social class patterns mirror previous research that has not found differences in educational background in caregiving by adult children in Sweden (Sarasa Urdiola and Billingsley 2008; von Essen and Svedberg 2020). However, our results revealed an interaction between the child’s gender and social class, suggesting that when studying child socioeconomic differences in intergenerational old age care one should account for the gender of the care providing adult child.

Our analyses returned two particularly important results. Firstly, daughters in the manual social class group were more often reported to be care providers than sons in the manual social class group, regardless of parental gender, household social class, and geographic proximity, whereas no gender differences could be seen among the other adult child social classes. Secondly, as the care intensity increased, so did the relative difference between daughters in the manual social class and all other children. Even if the difference was not significant across all caregiving groups, this still suggests that disparities reported among daughters are greater than between sons and daughters when accounting for social class. Our results confirm international findings (Cohen et al. 2019; Henz 2021) and complement the finding by Ulmanen and Szebehely (2015) in a Swedish context, who showed higher and increasing caregiving by daughters of parents with lower socioeconomic status. We found that the social class of the adult child is in itself an important factor for understanding gender differences in caregiving, regardless of parental social class. By considering care intensity and adding several parent–child characteristics, our finding also refines those of Winqvist (1999). We show that gender and socioeconomic differences among adult children exist even when controlling for geographic proximity where sons tend to live closer than daughters.

Reported gender differences among manual-working-class children could be explained through more traditional gender roles (West and Zimmerman 1987) compared to other social classes even in a relatively gender-equal country such as Sweden (EIGE 2021). One explanation could be that families of lower social classes have been shown to be more family-oriented than other social classes (Silverstein and Bengtson 1997). Another explanation suggests that women in manual occupations either work part-time or retire earlier than others with more time to spend on informal caregiving (Dentinger and Clarkberg 2002). However, we cannot exclude that daughters with manual occupations may decrease their working hours due to their parents’ care needs.

Informal caregiving can be a positive experience; however, negative consequences in terms of worse health, reduction, or termination of paid work and lower financial resources are common, especially among informal caregivers providing intensive care (Bastawrous et al. 2015; Johansson et al. 2022; Lilly et al. 2007). Furthermore, women are at higher risk of experiencing these negative consequences than men, even when accounting for gender differences in caregiving (Szebehely et al. 2014).

Our results support and exemplify research by Saraceno (2010), who investigated social inequalities in caregiving and care receiving from a bi-generational perspective. She argued that the structural features of public care support in any given country have a greater impact on family caregivers than care recipients from both a gender and social class perspective. When the overall coverage reduces, and the eligibility thresholds are relatively strict, older adults must have quite severe health problems to qualify for formal care. Consequently, the adult children of parents with less-severe health problems but who still need care will be affected (Rostgaard et al. 2022). However, this study shows that even when accounting for formal care received by parents, care received from children still vary depending on their gender and social class combined. It is also important to consider that daughters in manual occupations already face financial and social disadvantages, leaving them with fewer resources. In addition, these women are more likely to work in care-based occupations (Kjellsson 2021). Hence, social inequalities risk maintaining or even increasing inequalities in care receipt by older parents from adult children. Future research should monitor recent developments in unequal intergenerational care transfers in old age and try to understand how an adult child’s gender and social class influences any care decisions received and provided.

## Strengths and limitations

This study focuses on care reported and received by older parents in need from each individual adult child and therefore comes with both strengths and limitations. Assessing intergenerational transfers of resources may include risks of reporting bias from either generation and can vary depending on, e.g. type of support and differences in measurements. In a study from the USA by Lin and Wu (2017), adult child reports of time transfers provided to parents were found to be more reliable than that of parents when measured as a binary question and including children over 18 years and parents under 80 years. In another study from the USA, Kim et al. (2011), however, did not find any significant difference when measuring frequency of practical support while including children between the ages 40–60 and their parents 96 years and below. Bearing this in mind we consider the strengths of this study to include: (1) covering of the care received from parents in relation to all individual adult children which is a rare feature in previous studies (Lin 2017); (2) the inclusion of the oldest-old, which is a group with high care consumption, often underrepresented in informal care research; and (3) the ability to measure care intensity. To our knowledge, no other Swedish data provide the opportunity to consider these aspects when assessing informal care received by older parents from adult children.

The operationalisation of care intensity is an additional strength, measured by number of care tasks while considering type of care. By assessing care intensity in this way, we offer an alternative for when there is a lack of information about frequencies in caregiving. The list of care tasks included was not comprehensive; however, they covered a broad scope. This enabled us to assess both non-intensive and intensive caregiving, thereby capturing a more accurate picture of subgroup differences in caregiving (Szebehely 2005). The occupational grouping of adult children is a limitation. The non-manual social class category can include anything from less-qualified office staff to managing directors or professors. Results might have been more nuanced with more detailed social class categories. Given the sometime restricted knowledge parents had concerning their children’s occupation, this was, however, not possible leading to a potential underestimation of the results.

## Policy implications

This study challenges the idea that care of older adults in Sweden should be available for all when needed regardless of an individual’s resources. The results demonstrate that deficiencies in this egalitarian system have consequences for both older parents receiving care and their adult children. This is worrying, especially as the proportion of older adults in need of care will double over the coming decade in Sweden (National Board of Health and Welfare 2020), while family care has been increasing over time (von Essen and Svedberg 2020). Another issue relates to the sustainability of the welfare system; the informal caregiving obligations of women must be recognised, if they are to continue working in the welfare sector where both their taxes and labour contributions are needed to support the system. Policies should therefore consider care intensity and a *combination* of gender and socioeconomic status, while identifying ways of counteracting inequalities in intergenerational caregiving.

## Conclusions

Even in a strong welfare state and an equality-conscious country such as Sweden, older parents report a significant proportion of adult children providing them with informal care. Although reported to be generally less intensive, this care is unequally distributed among children. Daughters with manual occupations were reported to be the most common carers and are overrepresented in providing intensive care. These women are thereby at risk of experiencing adverse consequences in their lives. Hence, knowledge about levels and patterns of intergenerational care transfers in families of older adults has important implications on how to reduce care inequalities.

## References

[CR1] Andersson L-G, Erikson R, Wärneryd B (1981) Att beskriva den sociala strukturen: Utvärdering av 1974 års förslag till socio-ekonomisk indelning, vol 19. Statistisk tidskrift.

[CR2] Bastawrous M, Gignac MA, Kapral MK, Cameron JI (2015). Factors that contribute to adult children caregivers' well-being: a scoping review. Health Soc Care Community.

[CR3] Brandt M (2011). Intergenerational help and public assistance in Europe—a case of specialization?. SSRN Electron J.

[CR4] Carmichael F, Charles S, Hulme C (2010). Who will care? Employment participation and willingness to supply informal care. J Health Econ.

[CR5] Chan TW, Ermisch J (2015). Proximity of couples to parents: influences of gender, labor market, and family. Demography.

[CR6] Choi H, Schoeni RF, Langa KM, Heisler MM (2015). Spouse and child availability for newly disabled older adults: socioeconomic differences and potential role of residential proximity. J Gerontol B Psychol Sci Soc Sci.

[CR7] Cohen SA, Sabik NJ, Cook SK, Azzoli AB, Mendez-Luck CA (2019). Differences within differences: gender inequalities in caregiving intensity vary by race and ethnicity in informal caregivers. J Cross Cult Gerontol.

[CR8] Deindl C, Brandt M (2011). Financial support and practical help between older parents and their middle-aged children in Europe. Ageing Soc.

[CR9] Dentinger E, Clarkberg M (2002). Informal caregiving and retirement timing among men and women. J Fam Issues.

[CR10] EIGE (2021) European institute for gender equality. Gender equality index—index score for Sweden for the 2021 edition. https://eige.europa.eu/gender-equality-index/2021/SE

[CR11] Erikson R (1984). Social class of men, women and families. Sociology.

[CR12] Erikson R, Goldthorpe JH (1992). The constant flux: a study of class mobility in industrial societies.

[CR13] Fors S, Illinca S, Jull J, Kadi S, P Phillips S, Rodrigues R, Vafaei A, Zolyomi E, Rehnberg J (2022). Cohort-specific disability trajectories among older women and men in Europe 2004–2017. Eur J Ageing.

[CR14] Gautun H, Hagen K (2010). How do middle-aged employees combine work with caring for elderly parents?. Community Work Fam.

[CR15] Glaser K, Grundy E (2002). Class, caring and disability: evidence from the British Retirement Survey. Ageing Soc.

[CR16] Graubard BI, Korn EL (1999). Predictive margins with survey data. Biometrics.

[CR17] Grigoryeva A (2017). Own gender, sibling’s gender, parent’s gender. Am Sociol Rev.

[CR18] Henz U (2021). The ageing of parent carers: classed and gendered care-giving patterns at higher ages. Ageing Soc.

[CR19] Jegermalm M (2006). Informal care in Sweden: a typology of care and caregivers. Int J Soc Welfare.

[CR20] Jegermalm M, Grassman EJ (2012). Helpful citizens and caring families: patterns of informal help and caregiving in Sweden in a 17-year perspective. Int J Soc Welfare.

[CR21] Johansson MF, McKee KJ, Dahlberg L, Summer Meranius M, Williams CL, Marmstål Hammar L (2022). Negative impact and positive value of caregiving in spouse carers of persons with dementia in Sweden. Int J Environ Res Public Health.

[CR22] Kim K, Zarit SH, Eggebeen DJ, Birditt KS, Fingerman KL (2011). Discrepancies in reports of support exchanges between aging parents and their middle-aged children. J Gerontol Ser B.

[CR23] Kjellsson S (2021). Do working conditions contribute differently to gender gaps in self-rated health within different occupational classes? Evidence from the Swedish Level of Living Survey. PLoS ONE.

[CR24] Kridahl L, Duvander A-Z (2021). Are mothers and daughters most important? How gender, childhood family dissolution and parents’ current living arrangements affect the personal care of parents. Soc Sci.

[CR25] Lennartsson C (2001). Still in touch: family contact, activities and health among the elderly in Sweden.

[CR26] Lennartsson C, Agahi N, Hols-Salen L, Kelfve S, Kareholt I, Lundberg O, Parker MG, Thorslund M (2014). Data resource profile: the Swedish panel study of living conditions of the oldest old (SWEOLD). Int J Epidemiol.

[CR27] Li J, Song Y, Gu D, Dupre ME (2019). Formal and informal care. Encyclopedia of gerontology and population aging.

[CR28] Lilly MB, Laporte A, Coyte PC (2007). Labor market work and home care's unpaid caregivers: a systematic review of labor force participation rates, predictors of labor market withdrawal, and hours of work. Milbank Q.

[CR29] Lin I-F, Wu H-S (2017). Intergenerational transfer and reporting bias: an application of the MIMIC model. J. Gerontol. Ser. B.

[CR30] Litwak E (1985). Complementary roles for formal and informal support groups: a study of nursing homes and mortality rates. J Appl Behav Sci.

[CR31] Mood C (2010). Logistic regression: why we cannot do what we think we can do, and what we can do about it. Eur Sociol Rev.

[CR32] National Board of Health and Welfare (2020) *Anhöriga som vårdar eller stödjer närstående äldre personer* (2020-11-7045). Retrieved from Stockholm, Sweden. https://www.socialstyrelsen.se/globalassets/sharepoint-dokument/artikelkatalog/ovrigt/2020-11-7045.pdf

[CR33] Nilsen C, Agahi N, von Saenger I, Österman J, Rundgren ÅH, Lennartsson C (2019) Hur mår stockholmarna efter 65? Beskrivning av hälsa och levnadsvanor 2002–2018. https://aldrecentrum.se/wp-content/uploads/2020/06/2019_3-Hur-m%C3%A5r-stockholmarna-efter-65-beskrivning-av-h%C3%A4lsa-och-levnadsvanor-2002-2018.pdf

[CR34] Pettersson A, Malmberg G (2009). Adult children and elderly parents as mobility attractions in Sweden. Popul Space Place.

[CR35] Pillemer K, Suitor JJ (2013). Who provides care? A prospective study of caregiving among adult siblings. Gerontologist.

[CR36] Prop. 2008/09:82 (2008) Stöd till personer som vårdar eller stödjer närstående. Government Offices of Sweden. https://www.regeringen.se/contentassets/51fe0e3e78994715a9f58a7c43c46f4c/stod-till-personer-som-vardar-eller-stodjer-narstaende-prop.-20080982

[CR37] Qualls SH, Ferraro KF, Carr D (2021). Chapter 14—family caregiving. Handbook of aging and the social sciences.

[CR38] Rostgaard T, Jacobsen F, Kröger T, Peterson E (2022). Revisiting the Nordic long-term care model for older people—still equal?. Eur J Ageing.

[CR39] Saraceno C (2010). Social inequalities in facing old-age dependency: a bi-generational perspective. J Eur Soc Policy.

[CR40] Sarasa Urdiola S, Billingsley S (2008) Personal and household care giving for adult children to parents and social stratification. https://repositori.upf.edu/handle/10230/276

[CR41] Silverstein M, Bengtson VL (1997). Intergenerational solidarity and the structure of adult child-parent relationships in American families. Am J Sociol.

[CR42] Silverstein M, Gans D, Yang F (2006). Intergenerational support to aging parents. J Fam Issues.

[CR43] Sipilä J (2019). Social care services: the key to the Scandinavian welfare model.

[CR44] Svallfors S (2011). A bedrock of support? Trends in welfare state attitudes in Sweden, 1981–2010. Soc Pol Admin.

[CR45] Szebehely M (2005) Anhörigas betalda och obetalda äldreomsorgsinsatser*.* Forskarrapporter till Jämställdhetspolitiska utredningen SOU 2005: 66. https://www.regeringen.se/rapporter/2005/08/forskarrapporter-till-jamstalldhetspolitiska-utredningen/

[CR46] Szebehely M, Meagher G (2018). Nordic eldercare—weak universalism becoming weaker?. J Eur Soc Policy.

[CR47] Szebehely M, Trydegård G-B (2007). Omsorgstjänster för äldre och funktionshindrade: skilda villkor, skilda trender?. Socialvetenskaplig Tidskrift.

[CR48] Szebehely M, Ulmanen P, Sand A-B (2014) Att ge omsorg mitt i livet: hur påverkar det arbete och försörjning?. https://www.diva-portal.org/smash/get/diva2:684124/FULLTEXT01.pdf

[CR49] Szydlik M (2016). Sharing lives: adult children and parents.

[CR50] Tokunaga M, Hashimoto H (2017). The socioeconomic within-gender gap in informal caregiving among middle-aged women: evidence from a Japanese nationwide survey. Soc Sci Med.

[CR51] Tough H, Brinkhof MW, Siegrist J, Fekete C, SwiSCI Study Group (2019). Social inequalities in the burden of care: a dyadic analysis in the caregiving partners of persons with a physical disability. Int J Equity Health.

[CR52] Ulmanen P (2015) Omsorgens pris i åtstramningens tid. Stockholm university, Stockhom. http://su.diva-portal.org/smash/record.jsf?pid=diva2%3A858835&dswid=6081(150)

[CR53] Ulmanen P (2016). Kvinnors och mäns hjälp till sina gamla föräldrar – innehåll, omfattning och konsekvenser. Socialvetenskaplig Tidskrift.

[CR54] Ulmanen P (2022). Reversed socioeconomic pattern in the costs of caring regarding well-being and paid work among women in Sweden. Soc Pol Admin.

[CR55] Ulmanen P, Szebehely M (2015). From the state to the family or to the market? Consequences of reduced residential eldercare in Sweden. Int J Soc Welfare.

[CR56] van Groenou MB, Glaser K, Tomassini C, Jacobs T (2006). Socio-economic status differences in older people's use of informal and formal help: a comparison of four European countries. Ageing Soc.

[CR57] Verbakel E (2018). How to understand informal caregiving patterns in Europe? The role of formal long-term care provisions and family care norms. Scand J Public Health.

[CR58] von Essen J, Svedberg L (2020) Medborgerligt engagemang i Sverige 1992–2019. https://esh.diva-portal.org/smash/get/diva2:1470097/FULLTEXT01.pdf

[CR59] West C, Zimmerman DH (1987). Doing gender. Gender Soc.

[CR60] Williams R (2020) Adjusted predictions and marginal effects for multiple outcome models and commands (including ologit, mlogit, oglm, and gologit2). In: Handout, Notre Dame. University of Notre Dame

[CR61] Wimo A, Handels R, Elmståhl S, Fagerström C, Fratiglioni L, Isaksson U, Larsen O, Sanmartin Berglund J, Sjölund BM, Sköldunger A, Wahlberg M (2020) Informell och formell vård hos äldre personer i ordinärt boende – förändringar och samspel över tid 2001–2015 i SNAC projektet. https://www.snac-k.se/wp-content/uploads/2021/03/SNAC_Informell_formell_vard_2020.pdf

[CR62] Winqvist M (1999). Vuxna barn med hjälpbehövande föräldrar–en livsformsanalys (Adult children of parents in need of assistance–a life-mode analysis).

[CR63] Wolff JL, Mulcahy J, Huang J, Roth DL, Covinsky K, Kasper JD (2017). Family caregivers of older adults, 1999–2015: trends in characteristics, circumstances, and role-related appraisal. Gerontologist.

[CR64] Wong EL, Liao JM, Etherton-Beer C, Baldassar L, Cheung G, Dale CM, Flo E, Husebø BS, Lay-Yee R, Millard A, Peri KA (2020). Scoping review: intergenerational resource transfer and possible enabling factors. Int J Environ Res Public Health.

[CR65] Zimmerman E, Woolf SH (2014) Understanding the relationship between education and health. *NAM Perspectives*. 10.31478/201406a

